# Supplementary material for: Impact of integrative care on cardiovascular disease risk in newly diagnosed type 2 diabetes mellitus patients: A BI-VitalLife Cohort study
Source: PLoS One. 2024 May 29;19(5):e0302438. doi: 10.1371/journal.pone.0302438 (PMC11135683; doi:10.1371/journal.pone.0302438)
Supplement: S1 Table — (DOCX) [file pone.0302438.s002.docx]

**S2 Table.** Characteristics of the Participants before and after 1:4 and 1:2 Propensity Score Matching.

|  | Before Propensity Score Matching | | | After Propensity Score Matching (1:4) | | | After Propensity Score Matching (1:2) | | |
| --- | --- | --- | --- | --- | --- | --- | --- | --- | --- |
|  | Conventional Care | Integrative Care | p-value | Conventional Care | Integrative Care | p-value | Conventional Care | Integrative Care | p-value |
| N | 4,255 | 197 |  | 799 | 197 |  | 365 | 197 |  |
| Age at T2DM Diagnosis (years; Mean±SD) | 55.79 ± 12.45 | 51.54 ± 12.37 | <0.001 | 51.42 ± 12.05 | 51.54 ± 12.37 | 0.905 | 51.55 ± 12.05 | 51.54 ± 12.37 | 0.994 |
| Female (%) | 38.12 | 40.10 | 0.576 | 41.05 | 40.10 | 0.808 | 44.33 | 40.10 | 0.328 |
| Hemoglobin (g/dl) | 13.79 ± 1.83 | 14.02 ± 1.65 | 0.085 | 13.88 ± 1.77 | 14.02 ± 1.65 | 0.324 | 13.90 ± 1.76 | 14.02 ± 1.65 | 0.431 |
| RBC (x 10^6/ul) | 4.84 ± 0.70 | 5.00 ± 0.63 | 0.002 | 4.88 ± 0.69 | 5.00 ± 0.63 | 0.034 | 4.89 ± 0.70 | 5.00 ± 0.63 | 0.073 |
| MCH (pg) | 28.80 ± 3.53 | 28.57 ± 3.02 | 0.374 | 28.68 ± 3.41 | 28.57 ± 3.02 | 0.688 | 28.69 ± 3.24 | 28.57 ± 3.02 | 0.680 |
| MCHC (g/dl) | 33.44 ± 1.14 | 33.60 ± 1.16 | 0.046 | 33.44 ± 1.16 | 33.60 ± 1.16 | 0.073 | 33.46 ± 1.13 | 33.60 ± 1.16 | 0.144 |
| RDW (%) | 13.79 ± 1.90 | 13.54 ± 1.72 | 0.067 | 13.72 ± 1.83 | 13.54 ± 1.72 | 0.203 | 13.68 ± 1.68 | 13.54 ± 1.72 | 0.337 |
| WBC (x 10^3/ul) | 7.57 ± 3.07 | 6.99 ± 2.18 | 0.008 | 7.01 ± 2.20 | 6.99 ± 2.18 | 0.886 | 7.04 ± 2.32 | 6.99 ± 2.18 | 0.781 |
| Neutrophil (%) | 57.87 ± 12.28 | 56.59 ± 10.87 | 0.149 | 55.53 ± 11.11 | 56.59 ± 10.87 | 0.231 | 55.38 ± 10.49 | 56.59 ± 10.87 | 0.192 |
| Neutrophil (x 10^3/ul) | 4.56 ± 2.84 | 4.04 ± 1.78 | 0.011 | 3.99 ± 1.87 | 4.04 ± 1.78 | 0.710 | 3.99 ± 1.92 | 4.04 ± 1.78 | 0.779 |
| Lymphocyte (%) | 31.14 ± 10.84 | 32.51 ± 9.68 | 0.081 | 33.29 ± 10.07 | 32.51 ± 9.68 | 0.323 | 33.33 ± 9.68 | 32.51 ± 9.68 | 0.331 |
| Lymphocyte (x 10^3/ul) | 2.21 ± 0.94 | 2.20 ± 0.78 | 0.783 | 2.26 ± 0.80 | 2.20 ± 0.78 | 0.331 | 2.27 ± 0.81 | 2.20 ± 0.78 | 0.287 |
| Platelet (x 10^3/ul) | 253.89 ± 78.20 | 255.86 ± 62.52 | 0.727 | 253.02 ± 73.24 | 255.86 ± 62.52 | 0.616 | 255.42 ± 77.81 | 255.86 ± 62.52 | 0.945 |
| Creatinine (mg/dl) | 0.94 ± 0.59 | 0.88 ± 0.97 | 0.190 | 0.87 ± 0.36 | 0.88 ± 0.97 | 0.829 | 0.87 ± 0.39 | 0.88 ± 0.97 | 0.966 |
| EGFR (ml/min/1.73m2) | 92.20 ± 22.79 | 100.31 ± 20.44 | <0.001 | 97.12 ± 20.08 | 100.31 ± 20.44 | 0.047 | 96.31 ± 20.85 | 100.31 ± 20.44 | 0.027 |
| Cholesterol (mg/dl) | 197.34 ± 53.34 | 204.72 ± 56.08 | 0.058 | 199.77 ± 47.46 | 204.72 ± 56.08 | 0.207 | 199.56 ± 46.13 | 204.72 ± 56.08 | 0.233 |
| Age at CVD Diagnosis (years; Mean±SD) | 61.18 ± 11.90 (n=1,507) | 63.00 ± 12.34 (n=40) | 0.341 | 57.39 ± 11.06 (n=266) | 63.00 ± 12.34 (n=40) | 0.003 | 57.50 ± 11.43 (n=141) | 63.00 ± 12.34 (n=40) | 0.009 |
| CVD in 120 months (%) | 34.14 | 16.24 | <0.001 | 32.04 | 16.24 | <0.001 | 33.57 | 15.25 | <0.001 |
